# Suppression of autophagy by mycophenolic acid contributes to inhibition of HCV replication in human hepatoma cells

**DOI:** 10.1038/srep44039

**Published:** 2017-03-09

**Authors:** Shoucai Fang, Jinming Su, Bingyu Liang, Xu Li, Yu Li, Junjun Jiang, Jiegang Huang, Bo Zhou, Chuanyi Ning, Jieliang Li, Wenzhe Ho, Yiping Li, Hui Chen, Hao Liang, Li Ye

**Affiliations:** 1 Guangxi Key Laboratory of AIDS Prevention and Treatment & Guangxi Universities Key Laboratory of Prevention and Control of Highly Prevalent Disease, School of Public Health, Guangxi Medical University, Nanning 530021, Guangxi, China; 2 Guangxi Collaborative Innovation Center for Biomedicine, Life Sciences Institute, Guangxi Medical University, Nanning 530021, Guangxi, China; 3 Division of HIV/AIDS Control and Prevention, Guangxi Center for Disease Control and Prevention, Nanning 530021, Guangxi, China; 4 Department of Pathology and Laboratory Medicine, Temple University School of Medicine, Philadelphia, PA 19140, USA; 5 Institute of Human Virology and Key Laboratory of Tropical Disease Control of Ministry of Education, Zhongshan School of Medicine, Sun Yat-sen University, Guangzhou 510080, China; 6 Geriatrics Digestion Department of Internal Medicine, The First Affiliated Hospital of Guangxi Medical University, Nanning 530021, Guangxi, China

## Abstract

Previous studies have shown that mycophenolic acid (MPA) has an anti-HCV activity. However, the mechanism of MPA-mediated inhibition of HCV replication remains to be determined. This study investigated whether MPA has an effect on autophagy, a cellular machinery required for HCV replication, thereby, inhibits HCV replication in Huh7 cells. MPA treatment of Huh7 cells could suppress autophagy, evidenced by decreased LC3B-II level and conversion of LC3B-I to LC3B-II, decreased autophagosome formation, and increased p62 level compared to MPA-untreated cells. Tunicamycin treatment or HCV infection could induce cellular autophagy, however, MPA also exhibited its inhibitory effect on tunicamycin- or HCV infection-induced autophagy. The expression of three autophagy-related genes, Atg3, Atg5, and Atg7 were identified to be inhibited by MPA treatment. Over-expression of these genes could partly recover HCV replication inhibited by MPA; however, silencing their expression by siRNAs could enhance the inhibitory effect of MPA on HCV. Collectively, these results reveal that suppression of autophagy by MPA plays a role in its anti-HCV activity. Down-regulating the expression of three autophagy-related genes by MPA involves in its antiviral mechanism.

Hepatitis C virus (HCV) infection is a global public health problem, with 170 million infected individuals worldwide, which represents ~3% of the world’s population^1^,^2^. It is estimated 350 to 500 thousands deaths due to HCV-related hepatic diseases each year. The HCV epidemic can not be ignored in China where the estimated infection number is 5~10 million^3^. The end-stage hepatic diseases associated with HCV infection is a major reason of liver transplantation in the United States and Europe^4^, which has become the effective operation for treatment of end-stage hepatic diseases^5^. However, the recurrence of HCV infection is the most common and serious complication of liver transplant HCV-infected patients, in post-transplantation which occurs in 70~80% of recipients, of whom 10~21% develop fibrosis and cirrhosis^4^. To prevent post-transplant graft rejection, the immunosuppressiants have also been applied widely in liver transplantation. Acting as a double-edged sword, the immunosuppression may accelerate viral replication, resulting in progression of chronic hepatitis C to severe allograft, fibrosis and cirrhosis^6^.

Mycophenolate mofetil (MMF) has been considered to be an effective and safe immunosuppressive agent in organ transplantations^7^ and treatments of autoimmune diseases^8^. Compared with calcineurin inhibitors (Cyclosporine and FK506), MMF lacks the nephrotoxicity. It is often used as a substitute or combined agent of cyclosporine in organ transplantations as well as treatments for acute rejection^9^,^10^. The clinical effects of MMF on HCV recurrence or replication in liver transplantations are controversial, with some reports showing that MMF treatment could reduce HCV recurrence incidence, serum HCV viral load and/or HCV-related fibrosis after liver transplantation^11^,^12^,^13^; while others indicating no change or a slight increase in HCV viral load^14^,^15^,^16^,^17^,^18^. In contrast to inconsistent clinical results, a few *in vitro* studies consistently support that MMF or mycophenolic acid (MPA), the active metabolite of MMF, has potent antiviral activity against HCV infection^19^,^20^,^21^. The general antiviral mechanisms of MPA are believed to involve in two aspects, guanosine depletion and enhancement of interferon (IFN)-stimulated genes (ISGs) expression^21^,^22^. However, both mechanisms could not fully explain the inhibitory effect on HCV replication by MPA^22^,^23^, suggesting that other mechanisms be also involved.

In recent years, interrelationship between the cellular autophagy and HCV has been a hot area of research. HCV has the ability to induce autophagy; on the other hand, autophagy machinery plays an important role in HCV pathogenesis. A number of autophagy-related proteins, including Beclin 1, LC3, Atg4B, Atg5, Atg7 and Atg12, have been identified to be required for productive HCV infection^24^,^25^,^26^,^27^. These proteins are beneficial for HCV replication, through modulating the onset of translation of incoming HCV RNA or contributing to HCV particle assembly and/or secretion^24^,^25^,^26^,^27^,^28^. Therefore, inhibition of HCV-induced autophagy could be used as a strategy to block HCV infection or transmission. In this study, we investigated whether MPA blocks the autophagy in human hepatoma cells, thereby, inhibits HCV infection/replication in cells.

## Results

### MPA inhibits HCV JFH-1 replication in Huh7 cells

**Figure 1:**
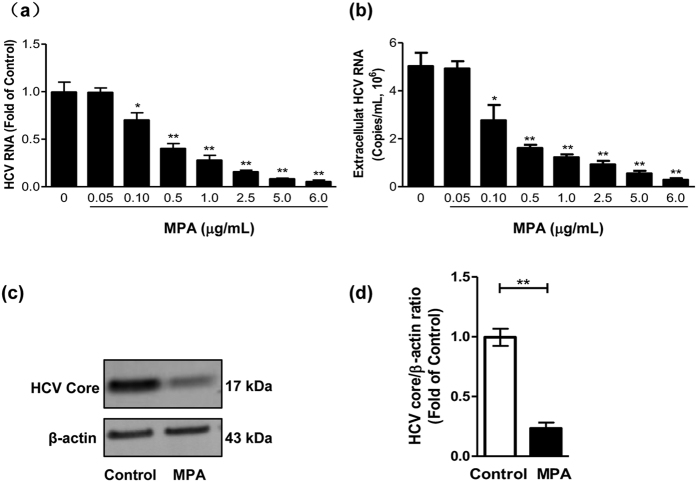
Inhibition of HCV replication by MPA treatment in Huh7 cells. Huh7 cells were infected with HCV JFH-1 at an MOI of 0.1. At day 3 postinfection, the infected cells were treated with MPA at indicated concentrations for 48 h. The cellular/extracellular RNA and cellular proteins were extracted for real-time RT PCR (**a**,**b**) and western blot analysis (**c**,**d**). (**a**) The levels of intracellular HCV RNA in MPA-treated or untreated control cells, with normalization to corresponding GAPDH mRNA level, are expressed as the fold of control (without MPA treatment, which was defined as 1). (**b**) The levels of extracellular HCV RNA in culture supernatants of MPA-treated or untreated control cells were measured and expressed as copies/mL. (**c**) The effect of MPA on HCV core expression in JFH-1 infected Huh7 cells. A representative western blot image shows the HCV core protein expression. (**d**) Quantitative assessment of HCV core protein expression in MPA-treated or untreated Huh7 cells. The densitometric intensities of HCV core and β-actin bands were quantified by image J software. The relative HCV core/β-actin ratios were calculated and shown as the fold of control (without MPA treatment, which was defined as 1). The data shown in Fig. 1a,b and d are the mean ± SD of the results of three independent experiments. The *p* value was calculated by Student’s *t*-test (*p < 0.05, **p < 0.01).

MTS data show that there is no cytotoxic effect of MPA on Huh7 cells when its concentration is at 6 μg/mL or lower (Supplementary Fig. 1). Thus, the experiments in this study used the MPA concentrations within a range of 1 μg/mL to 5 μg/mL, which is consistent with clinically relevant concentrations. The liver transplant recipients receiving MMF had serum peak levels ranging from 0.6 μg/mL to 11.5 μg/mL, and trough levels average around 3 μg/mL^29^,^30^. As shown in Fig. 1, MPA treatment of HCV JFH-1-infected Huh7 cells significantly inhibited HCV RNA expression at both intracellular and extracellular levels (Fig. 1a and b). The inhibition concentration of MPA is in the range of 0.1 μg/mL to 6 μg/mL, and the inhibitory effect of MPA on HCV replication is in a dose-dependent manner. The inhibition of HCV replication by MPA treatment was also confirmed by western blot examining HCV core protein expression in Huh7 cells. As demonstrated in Fig. 1c and d, HCV core protein expression in MPA-treated cells was significantly lower than that in control cells.

### MPA treatment suppresses cellular autophagy in Huh7 cells

To examine whether MPA affects cellular autophagy in Huh7 cells, the relative levels of LC3B-II and p62 were first analyzed by western blot. LC3, a cytosolic ubiquitin-like protein, has two forms, LC3-I and LC3-II. During autophagy, the soluble form of LC3 (LC3-I) is converted to a lipidated form (LC3-II), which is associated with autophagosomal membranes. Thus, the level of LC3B-II is often used as a marker of autophagy. However, an increase (or a decrease) in LC3B-II level could be caused by either an autophagic flux induction (or suppression) or an inhibition (or a induction) of autophagosome clearance. Therefore, cells were also treated with a late stage autophagy inhibitor Bafilomycin A1 (Baf A1) to block the fusion of autophagosomes with lysosomes and to prevent lysosomal degradation, to clarify two different mechanisms. Accumulation of p62, was additionally a measure of autophagic flux. P62, a cargo receptor for autophagic degradation of ubiquitinated targets that binds directly to LC3-II, is exclusively degraded during autophagy. Thus, increased p62 levels could be observed when autophagy is inhibited, and decreased levels when autophagy is induced.

**Figure 2:**
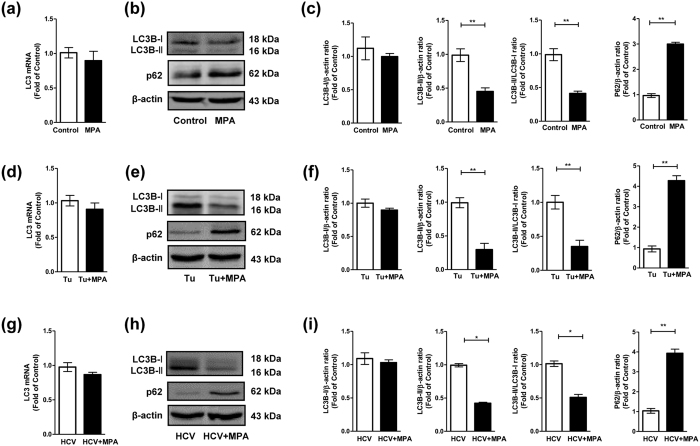
The effects of MPA treatment on LC3 and p62 levels in Huh7 cells, tunicamycin(Tu)-treated, or HCV-infected Huh 7 cells in the absence of Bafilomycin A1 (Baf A1). (**a**–**c**) Huh7 cells were treated or untreated with MPA (5 μg/mL) for 24 h; (**d**–**f)** Huh7 cells treated with tunicamycin (5 μg/mL) were simultaneously treated or untreated with MPA (5 μg/mL) for 24 h; (**g**–**i**) Huh 7 cells were infected with HCV JFH-1 at an MOI of 0.1. At day 3 postinfection, the HCV-infected cells were treated or untreated with MPA (5 μg/mL) for 24 h. After MPA treatment, the cellular RNA and proteins were extracted for real time RT PCR and western blot analysis. (**a**,**d**,**g**) The levels of intracellular LC3 mRNA in MPA-treated or untreated cells under different conditions, with normalization to corresponding GAPDH mRNA level, are expressed as the fold of control (without MPA treatment under different conditions, which was defined as 1, respectively; in Huh 7 cells, Tu-treated, or HCV-infected cells, the control group is MPA untreated cells, MPA untreated Tu-treated cells, or MPA untreated HCV-infected cells, respectively). (**b**,**e**,**h**) Representative western blot images show LC3B-I, LC3B-II, p62 protein levels in MPA-treated or untreated cells under different conditions. (**c**,**f**,**i**) Quantitative assessment of LC3B-I, LC3B-II, LC3B-II/LC3B-I, and p62 at protein level in MPA-treated or untreated cells under different conditions. The densitometric intensities of LC3B-I, LC3B-II, p62 and β-actin bands were quantified by image J software. The relative LC3B-I/β-actin, LC3B-II/β-actin, LC3B-II/LC3B-I, and p62/β-actin ratios were calculated and shown as the fold of control (without MPA treatment under different conditions, which was defined as 1, respectively). The data shown in Fig. 2a,c,d,f,g,i, are the mean ± SD of the results of three independent experiments. The *p* value was calculated by Student’s *t*-test (*p < 0.05, **p < 0.01).

In the absence of BafA1, at LC3 mRNA level, no significant effect (*p* > 0.05) was observed in MPA-treated cells compared to control cells (Fig. 2a). Accordingly, no significant effect (*p* > 0.05) was observed in LC3B-I level between MPA-treated and control cells (Fig. 2b,c). However, we observed a significant impact of MPA on LC3B-II level in Huh7 cells. The level of LC3B-II as well as conversion of LC3B-I to LC3B-II in MPA-treated cells significantly decreased compared to those in untreated control cells (Fig. 2b,c). Meanwhile, higher level of p62 was observed in MPA-treated cells (Fig. 2b,c). Therefore, in the absence of Baf A1, the decreased level of LC3B-II and conversion of LC3B-I to LC3B-II as well as increased level of p62 in MPA-treated cells imply that MPA treatment may suppress cellular autophagy (Fig. 2a–c).

**Figure 3:**
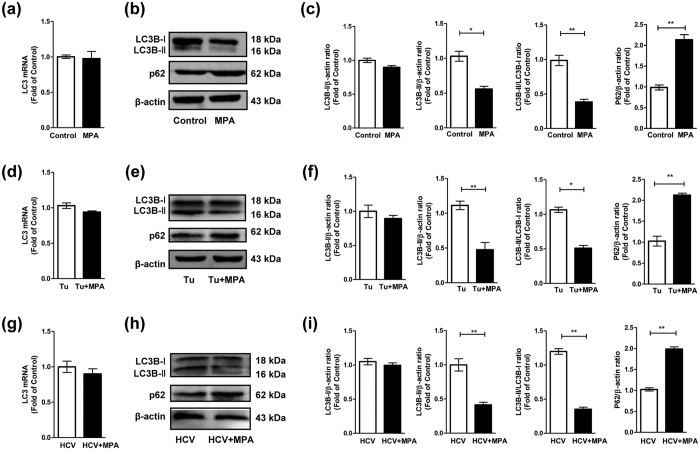
The effects of MPA treatment on LC3 and p62 levels in Huh7 cells, tunicamycin(Tu)-treated, or HCV-infected Huh 7 cells in the presence of Bafilomycin A1 (Baf A1). After 2 h pre-incubation with the autophagy inhibitor Baf A1 (5 nM), (**a**–**c**) Huh7 cells were treated or untreated with MPA (5 μg/mL) for 24 h; (**d**–**f**) Huh7 cells treated with tunicamycin (5 μg/mL) were simultaneously treated or untreated with MPA (5 μg/mL) for 24 h; (**g**–**i**) Huh 7 cells were infected with HCV JFH-1 at an MOI of 0.1. At day 3 postinfection, the HCV-infected cells were treated or untreated with MPA (5 μg/mL) for 24 h. After MPA treatment, the cellular RNA and proteins were extracted for real time RT PCR and western blot analysis. (**a**,**d**,**g**) The levels of intracellular LC3 mRNA in MPA-treated or untreated cells under different conditions, with normalization to corresponding GAPDH mRNA level, are expressed as the fold of control (without MPA treatment under different conditions, which was defined as 1, respectively; in Huh 7 cells, Tu-treated, or HCV-infected cells, the control group is MPA untreated cells, MPA untreated Tu-treated cells, or MPA untreated HCV-infected cells, respectively). (**b**,**e**,**h**) Representative western blot images show LC3B-I, LC3B-II, p62 protein levels in MPA-treated or untreated cells under different conditions. (**c**,**f**,**i**) Quantitative assessment of LC3B-I, LC3B-II, LC3B-II/LC3B-I, and p62 at protein level in MPA-treated or untreated cells under different conditions. The densitometric intensities of LC3B-I, LC3B-II, p62 and β-actin bands were quantified by image J software. The relative LC3B-I/β-actin, LC3B-II/β-actin, LC3B-II/LC3B-I, and p62/β-actin ratios were calculated and shown as the fold of control (without MPA treatment under different conditions, which was defined as 1, respectively). The data shown in Fig. 3a,c,d,f,g,i, are the mean ± SD of the results of three independent experiments. The *p* value was calculated by Student’s *t*-test (*p < 0.05, **p < 0.01).

In the presence of BafA1, at LC3 mRNA level or at LC3B-I protein level, no significant effect (*p* > 0.05) was observed in MPA-treated cells compared to control cells (Fig. 3a–c). However, decreased level of LC3B-II and conversion of LC3B-I to LC3B-II as well as increased level of p62 were observed in MPA-treated cells (Fig. 3b,c), which are similar to those observed in the absence of BafA1. Therefore, these results indicate that the decrease in LC3B-II level and conversion of LC3B-I to LC3B-II caused by MPA treatment is due to suppression of autophagy but not enhancement of autophagosome clearance.

### MPA treatment suppresses tunicamycin- or HCV- induced autophagy in Huh7 cells

We then examined the effects of MPA on LC3 and p62 levels under the conditions of tunicamycin (a known inducer of autophagy) treatment or HCV infection. In the absence of Baf A1, we observed that tunicamycin treatment or HCV infection could induce cellular autophagy, evidenced by significantly increased levels of LC3B-II and increased conversion of LC3B-I to LC3B-II, as well as decreased levels of p62 (Fig. 2e vs. 2b; Fig. 2h vs. 2b). Similar to its effect on cells without tunicamycin treatment/HCV infection (Fig. 2a–c), MPA treatment has little effect on LC3 mRNA expression as well as LC3B-I protein level under the condition of tunicamycin treatment (Fig. 2d–f) or HCV infection (Fig. 2g–i). However, MPA treatment could significantly decrease tunicamycin- or HCV- induced LC3B-II level and conversion of LC3B-I to LC3B-II (Fig. 2e,f,h,i). Under MPA treatment, accumulation of p62 was also observed in tunicamycin-treated or HCV-infected cells (Fig. 2e,f,h,i). In the presence of Baf A1, similar results (Fig. 3) were observed as those in the absence of Baf A1 (Fig. 2). Tunicamycin treatment or HCV infection could increase levels of LC3B-II and conversion of LC3B-I to LC3B-II, and decrease p62 levels (Fig. 3e vs. 3b; Fig. 3h vs. 3b). MPA treatment has little effect on LC3 mRNA expression as well as LC3B-I protein level under the condition of tunicamycin treatment (Fig. 3d–f) or HCV infection (Fig. 3g–i), while MPA treatment could significantly decrease tunicamycin- or HCV- induced LC3B-II level and conversion of LC3B-I to LC3B-II, and increase the levels of p62 (Fig. 3e,f,h,i) that were decreased by tunicamycin treatment or HCV infection (Fig. 3e vs. 3b; Fig. 3h vs. 3b). These results indicate that MPA treatment suppresses tunicamycin- or HCV- induced autophagy in Huh7 cells.

### MPA treatment inhibits tunicamycin- or HCV- induced autophagosome formation

**Figure 4:**
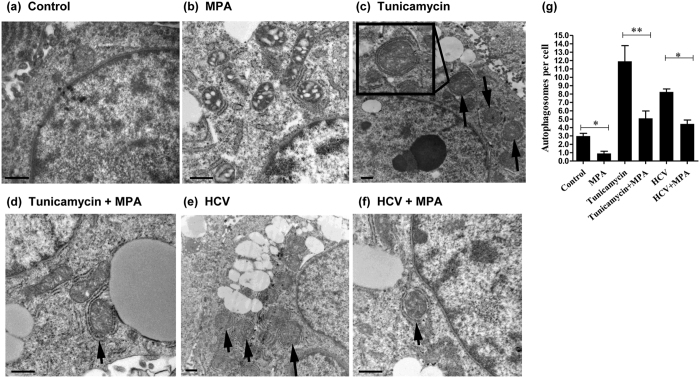
The effects of MPA treatment on autophagosome formation in Huh7 cells under different conditions. After 2 h pre-incubation with the autophagy inhibitor bafilomycin A1 (5 nM), MPA-treated and MPA-untreated Huh7 cells were subjected to ultrastructural analysis by transmission electron microscopy (TEM). (**a**,**b**) Huh7 cells without HCV infection/tunicamycin treatment were treated or untreated with MPA (5 μg/mL) for 24 h. (**c**,**d**) The tunicamycin-treated (5 μg/mL) cells were simultaneously treated or untreated with MPA (5 μg/mL) for 24 h. (**e**,**f**) Huh7 cells were infected with HCV JFH-1 at an MOI of 0.1. At day 3 postinfection, the HCV-infected cells were treated or untreated with MPA (5 μg/mL) for 24 h. (**g**) The counts of autophagosomal structures per cell were determined in MPA-treated or untreated cells under different conditions (Twenty cells were counted per experiment). The *p* value was calculated by Student’s *t*-test (*p < 0.05, **p < 0.01). Arrows indicate double-membrane vacuolar structures. Scale bars: 500 nm.

To confirm the findings based on LC3B-II and p62 levels, the autophagic activity was measured by the formation of autophagosomes under transmission electron microscopy (TEM). The closed double-membraned vesicles (Fig. 4, indicated by the arrows) with a diameter of 300 to 900 nm, resembling autophagic vesicles, were detected under different conditions (in the presence of bafilomycin A1). Few double-membraned vesicles were observed in control cells (without MPA/tunicamycin/HCV infection) (Fig. 4a,g) and less were observed in MPA-treated cells (Fig. 4b,g). Under the conditions of tunicamycin treatment or HCV infection, more double-membraned vesicles were observed (Fig. 4c,e,g), indicating that tunicamycin treatment or HCV infection could induce formation of autophagosomes. However, when tunicamycin-treated or HCV-infected cells were treated by MPA simultaneously, the significant diminution the double-membraned vesicles showing that MPA treatment inhibited the autophagosome formation induced by tunicamycin treatment or HCV infection (Fig. 4d,f,g).

### The effects of MPA treatment on the expression of autophagy-related genes

**Figure 5:**
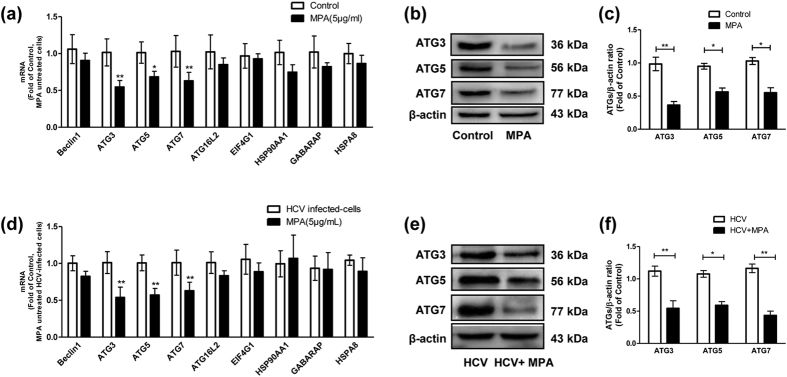
The effects of MPA treatment on the expression of autophagy-related genes (ATGs) in Huh7 cells or HCV-infected cells. (**a**–**c**) Huh7 cells were treated or untreated with MPA (5 μg/mL) for 24 h; (**d**–**f)** Huh 7 cells were infected with HCV JFH-1 at an MOI of 0.1. At day 3 postinfection, the HCV-infected cells were treated or untreated with MPA (5 μg/mL) for 24 h. After MPA treatment, the cellular RNA and proteins were extracted for real time RT PCR and western blot analysis. (**a**,**d**) The mRNA levels of ATGs (Beclin1, ATG3, ATG5, ATG7, ATG16L2, EIF4G1, GABARAP, HSP90AA1, and HSPA8) in MPA-treated or untreated cells under different conditions, with normalization to corresponding GAPDH mRNA level, are expressed as the fold of control (without MPA treatment under different conditions, which was defined as 1, respectively; in Huh 7 cells or HCV-infected cells, the control group is MPA untreated cells or MPA untreated HCV-infected cells, respectively). (**b**,**e**) Representative western blot images show ATG3, ATG5 and ATG7 protein levels in MPA-treated or untreated cells under different conditions. (**c**,**f**) Quantitative assessment of ATG3, ATG5 and ATG7 at protein level in MPA-treated or untreated cells under different conditions. The densitometric intensities of ATG3, ATG5, ATG7 and β-actin bands were quantified by image J software. The relative ATG3/β-actin, ATG5/β-actin, ATG7/β-actin ratios were calculated and shown as the fold of control (without MPA treatment under different conditions, which was defined as 1, respectively). The data shown in Fig. 5a,c,d,f are the mean ± SD of the results of three independent experiments. The *p* value was calculated by Student’s *t*-test (*p < 0.05, **p < 0.01).

Given that MPA treatment resulted in suppression of autophagy in Huh7 cells, we further investigated the autophagy-related genes expression profile under MPA treatment using Autophagy PCR Array. The changes of genes at least 2-fold regulated expression are shown in Table 1. In HCV-uninfected Huh7 cells, the expression of six genes, including ATG3, ATG5, ATG7, ATG16L2, EIF4G1, and GABARAP was down-regulated by MPA treatment. In HCV JFH-1-infected Huh7 cells, the expression of seven genes, including ATG3, ATG5, ATG7, Beclin 1, EIF4G1, HSP90AA1, and HSPA8 was down-regulated by MPA treatment. We then used the real time RT-PCR to validate the changed expression of autophagy-related genes. As shown in Fig. 5, the expression of three genes, ATG3, ATG5 and ATG7, was found to be down-regulated at mRNA level by MPA treatment, either in HCV-uninfected cells (Fig. 5a) or in HCV-infected cells (Fig. 5d). The effects of MPA treatment on expression of other autophagy-related genes were no longer observed (Fig. 5a,d). Western blot analysis was performed to further confirm the changes of ATG3, ATG5 and ATG7 expression caused by MPA treatment. The results show that the protein levels of ATG3, ATG5 and ATG7 in MPA-treated cells were significantly lower than those in MPA-untreated cells, either in HCV-infected or in HCV-uninfected Huh7 cells (Fig. 5b,c,e,f).

### Overexpression of ATG3, ATG5 or ATG7 weakens the inhibitory effect of MPA on HCV replication

**Figure 6:**
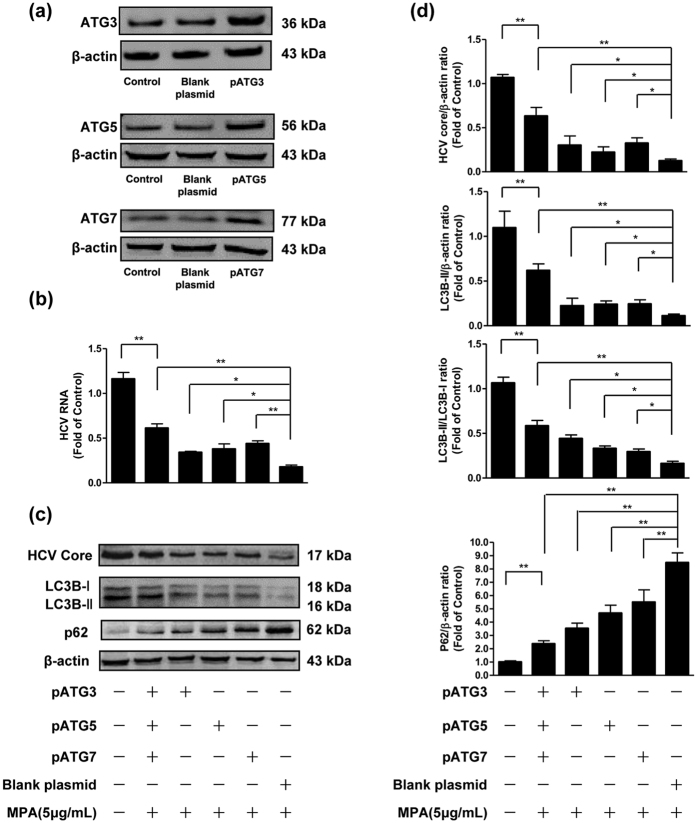
The effects of overexpression of ATG3, ATG5 or ATG7 on the inhibitory effect of MPA on HCV replication. Huh7 cells were infected with HCV JFH-1 at an MOI of 0.1. At day 3 postinfection, Huh7 cells were transfected with plasmids pCMV-myc-Atg3, pCMV-myc-Atg5 and/or pCMV-myc-Atg7, and the transfection maintained for 48 h. (**a**) Overexpression efficiency of pATG3, pATG5, and pATG7 were determined by western blot. (**b**–**d**) MPA (5 μg/mL) was added to cultures of plasmid-transfected cells and the treatment was maintained for 48 h. The cellular RNA and proteins were extracted for real-time RT-PCR and western blot analysis. (**b**) The effects of overexpression of ATG3, ATG5, ATG7 on HCV RNA expression. The levels of intracellular HCV RNA in Huh7 cells, with normalization to corresponding GAPDH mRNA level, are expressed as the fold of control (without MPA treatment/plasmid transfection, which was defined as 1). (**c**) A representative western blot image shows HCV core, LC3B-I, LC3B-II, p62 protein levels in Huh7 cells. (**d**) Quantitative assessment of HCV core, LC3B-II, LC3B-II/LC3B-I, p62 at protein level. The densitometric intensities of HCV core, LC3B-I, LC3B-II, p62, β-actin bands were quantified by image J software. The relative HCV core/β-actin, LC3B-II/β-actin, LC3B-II/LC3B-I, and p62/β-actin ratios were calculated and shown as the fold of control (without MPA treatment/plasmid transfection, which was defined as 1). The data shown in Fig. 6b,d are the mean ± SD of the results of three independent experiments. The *p* value was calculated by Student’s *t*-test (*p < 0.05, **p < 0.01).

Next, we investigate whether the over-expressed ATG3, ATG5 or ATG7 alters MPA-modulated inhibition of HCV (in the absence of Baf A1). We examined the levels of HCV RNA and HCV core protein when plasmids ATG3, ATG5 or ATG7 were transfected alone or together to MPA-treated HCV-infected Huh7 cells. Overexpression efficiency of pATG3, pATG5 and pATG7 in Huh7 cells was confirmed by western blot. Increased expression of ATG3, ATG5 or ATG7 was observed in pATG-transfected cells (Fig. 6a). As shown in Fig. 6, either at HCV RNA level or at HCV core protein level, overexpression of ATG3, ATG5 or ATG7 could restore HCV replication partially, evidenced by the increased levels of HCV RNA (Fig. 6b) or core protein expression (Fig. 6c,d) in pATG-transfected cells compared to MPA-treated cells with blank plasmid transfection. Co-transfection of plasmids ATG3, ATG5 or ATG7 had the highest recovery effect on HCV replication, with levels of HCV RNA (Fig. 6b) or core protein expression (Fig. 6c,d), reaching to 60~70% of those in MPA-untreated cells. We also examined the autophagic flux when the cells were transfected with pATGs. Increased LC3B-II levels and conversion of LC3B-I to LC3B-II were observed in pATG-transfected cells, compared to MPA-treated cells with blank plasmid transfection (Fig. 6c,d). On the contrary, decreased p62 levels were observed (Fig. 6c,d). These results indicate that overexpression of ATG3, ATG5 or ATG7 enhances cellular autophagy, along with partially restores HCV replication inhibited by MPA treatment.

### Silencing expression of ATG3, ATG5 or ATG7 enhances the inhibitory effect of MPA on HCV replication

**Figure 7:**
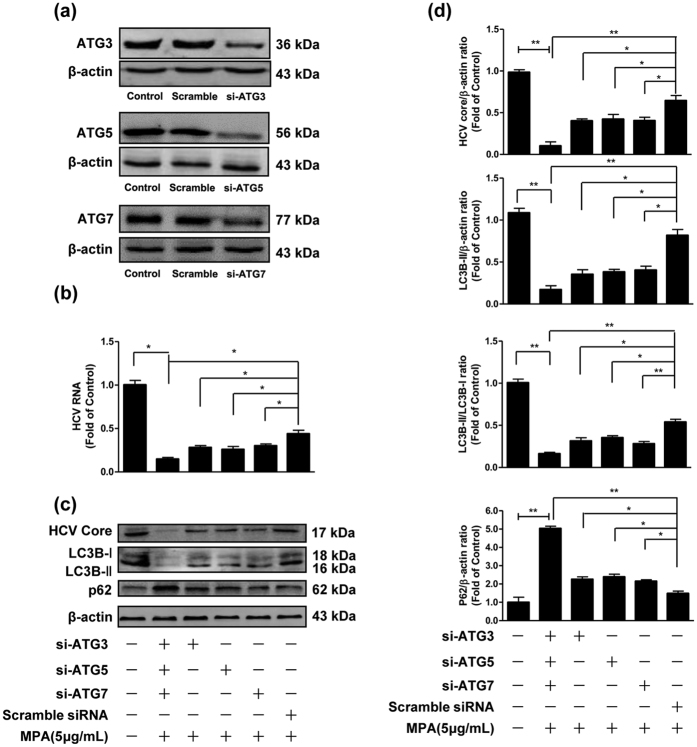
The effects of silencing expression of ATG3, ATG5 or ATG7 on the inhibitory effect of MPA on HCV replication. Huh7 cells were infected with HCV JFH-1 at an MOI of 0.1. At day 3 postinfection, Huh7 cells were transfected with specific siRNAs against ATG3, ATG5 or ATG7, and the transfection maintained for 48 h. (**a**) Silencing expression efficiency of si-ATG3, si-ATG5, and si-ATG7 were determined by western blot. (**b**–**d**) MPA (5 μg/mL) was added to cultures of siRNA-transfected cells and the treatment was maintained for 48 h. The cellular RNA and proteins were extracted for real-time RT-PCR and western blot analysis. (**b**) The effects of silencing expression of ATG3, ATG5, ATG7 on HCV RNA expression. The levels of intracellular HCV RNA in Huh7 cells, with normalization to corresponding GAPDH mRNA level, are expressed as the fold of control (without MPA treatment/siRNA transfection, which was defined as 1). (**c**) A representative western blot image shows HCV core, LC3B-I, LC3B-II, p62 protein levels in Huh7 cells. (**d**) Quantitative assessment of HCV core, LC3B-II, LC3B-II/LC3B-I, p62 at protein level. The densitometric intensities of HCV core, LC3B-I, LC3B-II, p62, β-actin bands were quantified by image J software. The relative HCV core/β-actin, LC3B-II/β-actin, LC3B-II/LC3B-I, and p62/β-actin ratios were calculated and shown as the fold of control (without MPA treatment/siRNA transfection, which was defined as 1). The data shown in Fig. 7b,d are the mean ± SD of the results of three independent experiments. The *p* value was calculated by Student’s *t*-test (*p < 0.05, **p < 0.01).

We also investigated the role of ATG3, ATG5 or ATG7 in MPA-modulated inhibition of HCV replication through silencing their expression via transfection of specific siRNAs against ATG3, ATG5 or ATG7 (in the absence of Baf A1). Silencing efficiency of si-ATG3, si-ATG5 or si-ATG7 in Huh7 cells was confirmed by western blot. Decreased expression of ATG3, ATG5 or ATG7 was observed in siRNA-transfected cells (Fig. 7a). As shown in Fig. 7, MPA-treated cells with silencing expression of ATG3, ATG5 or ATG7 further decreased HCV expression either at HCV RNA level (Fig. 7b) or at HCV core protein level (Fig. 7c,d), compared to the MPA-treated cells transfected with scrambled siRNA. Furthermore, co-transfection of three siRNAs had the highest contributable effect on inhibitory activity of MPA on HCV replication (Fig. 7b,c,d). The autophagic flux was also investigated when the cells were transfected siRNAs. Decreased LC3B-II level and conversion of LC3B-I to LC3B-II were observed in siRNA-transfected cells, compared to MPA-treated cells transfected with scrambled siRNA (Fig. 7c,d), while increased p62 levels were observed (Fig. 7c,d). These results indicate that silencing expression of ATG3, ATG5 or ATG7 suppresses cellular autophagy, along with enhances inhibitory effect of MPA on HCV replication.

## Discussion

In addition to its immunosuppression, MPA has been shown to have a broad antiviral activity against various viruses, including HCV, West Nile^21^,^31^, yellow fever^32^, and Chikungunya^33^ viruses. MPA has been suggested as a possible antiviral agent because of its ribavirin-like effects^34^. Ribavirin, which is used with pegylated IFN-α for clinical treatment of HCV, and MPA are the two representative inosine monophosphate dehydrogenase (IMPDH) inhibitors. The antiviral effects of MPA against the flaviviruses are generally believed to be due to its inhibition of IMPDH, as supplementation by exogenous guanosine nearly completely restores replication^21^,^31^,^32^,^33^. However, supplementation of guanosine has only little effect or partial action on the inhibition of HCV replication by MPA^19^,^21^,^35^, suggesting other mechanisms be also involved. A recent study shows that HCV infection through inducing an autophagy response to impair the anti-HCV activity of ribavirin^36^, which sparks our interest in investigation of relationship among MPA, HCV infection, and cellular autophagy.

Cellular autophagy’s primary function is to maintain energy homeostasis and nutrient balance during stressful conditions. It also plays a diverse role in host defense against invading pathogens^37^. HCV can subvert the autophagic pathway in favor of its own replication. In this study, we observed that HCV infection could induce autophagy, evidenced by increased LC3B-II level, conversion of LC3B-I to LC3B-II, and autophagosome formation as well as decreased p62 level (Figs 2 and 3), which is consistent with several recent findings that the accumulation of autophagy vesicles increased in HCV-infected cells^38^,^39^,^40^. Furthermore, our preliminary data show that HCV infection could up-regulate LC3, Beclin1, ATG3, ATG5, ATG7 and GABARAP expression at mRNA level (Supplementary Fig. 2), which is similar to the results of Sir *et al*.^41^, indicating the close relationship between HCV and autophagy. MPA treatment, however, could suppress HCV-induced autophagy, as evidenced by decreased LC3B-II level and conversion of LC3B-I to LC3B-II, decreased autophagosome formation, and increased level of p62, compared to MPA-untreated HCV-infected cells (Figs 2, 3, 4). Of course, one possibility for this result is that MPA firstly inhibits HCV replication through other antiviral mechanism(s), thereby suppresses HCV-induced autophagy. However, either in HCV-uninfected cells or in tunicamycin-treated cells, MPA exhibited its inhibitory effect on autophagy (Figs 2, 3, 4), showing that MPA does have the ability to directly inhibit cellular autophagy, not only simply through inhibition of HCV replication. In this study, tunicamycin treatment is used as positive control for observation of autophagy, which is a known autophagy inducer. It can cause accumulation of unfolded proteins in cell endoplasmic reticulum (ER) and induces ER stress and autophagy, which is similar to the mechanism of HCV-induced autophagy^42^,^43^.

Our mechanism results also confirmed the effect of MPA on autophagy. Three autophagy-related genes, ATG3, ATG5 and ATG7, were identified and verified to be down-regulated by MPA treatment (Table 1, Fig. 5). Overexpression of ATG3, ATG5 and ATG7 alone or together could recover the MPA-inhibited HCV replication to a certain degree (Fig. 6); whereas silencing their expression could enhance the inhibitory effect of MPA on HCV replication (Fig. 7). ATG3, ATG5 and ATG7 are important regulators of cellular autophagy. ATG3 acts a key factor of autophagy which can promote the combination of ATG8 and phosphatidyl ethanolamine (PE). Simultaneously, ATG3 participates in each stage of autophagosome formation and the binding of ATG5 and ATG12^44^,^45^. ATG5 is also a necessary factor for autophagy, which locates on the surface of pre-autophagosomal structure (PAS) via the combination of ATG12 and ATG16. The complex can promote the formation of autophagic vacuoles and promote LC3 to be present to autophagic vacuoles^41^. It has been reported that knockout of ATG5 could decline the production of HCV viral particles in infected Huh7.5 cells^46^. ATG7 plays a role in the early complex of autophagy and the formation of autophagic vacuoles through combining LC3 protein. Silencing expression of ATG7 could reduce cellular autophagy and lead to inhibition of HCV replication in infected hepatocytes^27^. Considering the important role of ATG3, ATG5 and ATG7 in cellular autophagy, our finding that MPA down-regulates their expression provides direct evidence that suppression of autophagy by MPA contributes its inhibitory effect on HCV replication.

Except their role in autophagy, some autophagy-related genes could directly involve in HCV replication. For example, ATG5 could combine with HCV RNA polymerase (NS5B) and promote the formation of viral complex as to translate of HCV RNA and launch the HCV replication^25^. Thus, the suppression of ATG5 expression by MPA may further contribute to inhibition of HCV replication through decrease of ATG5-NS5B interaction. More interestingly, ATG5 could also play a negative role in regulation of HCV pathogen-associated molecular pattern (PAMP)–mediated cytoplasmic retinoic acid–inducible gene I (RIG-I) signaling and type I interferon (IFN)–mediated antiviral responses. Recent studies demonstrate that Atg5-Atg12 conjugate negatively regulates the type I IFN pathway by direct combination to the RIG-I and IPS-1^26^,^47^,^48^. Several previous publications have highlighted that MPA can act in synergy with IFN-α on HCV replication^21^ and augment interferon-stimulated genes (ISGs) expression^22^. However, the mechanism(s) is still unclear. Our finding of down-regulation of ATG5 expression by MPA provides a meaningful clue for the further study on this interesting issue.

In summary, in the current study we demonstrated that MPA significantly inhibited HCV replication. The antiviral activity of MPA was found to be partly dependent on its suppression effect on autophagy and involved in the down-regulation of ATG3, ATG5 and ATG7 expression. These findings reveal a novel mechanism of anti-HCV activity of MPA and the relationship between MPA and HCV infection in the context of cellular autophagy, indicating blocking autophagy is an effective strategy for the treatment of HCV.

## Materials and Methods

### Reagents

Mycophenolic acid (MPA), tunicamycin, and bafilomycin A1 were purchased from Sigma-Aldrich China (Shanghai, China). The primary antibodies, including rabbit anti-β-actin, mouse anti-LC3 antibody, rabbit anti-p62 antibody, mouse anti-ATG3 antibody, mouse anti-ATG5 antibody, mouse anti-ATG 7 antibody and the secondary antibodies, including goat anti-mouse IgG antibody, goat anti-rabbit IgG antibody were obtained from Sigma-Aldrich China (Shanghai, China). Mouse anti-HCV core antibody was purchased from Thermo Fisher Scientific China (Shanghai, China). Plasmids pCMV-myc-Atg3, pCMV-myc-Atg5, pCMV-myc-Atg7 were obtained from Addgene (www.addgene.org, MA, USA).

### Cell culture, HCV JFH-1 infection, and transfection

Human hepatoma cell line Huh7 and HCV JFH-1 strain were kindly provided by Dr. Wenzhe Ho (Temple University School of Medicine, USA). Huh7 cells were maintained in Dulbecco’s modified Eagle’s medium (DMEM) supplemented with 10% fetal bovine serum (FBS), 100 U/mL penicillin, and 100 μg/mL streptomycin at 37 °C with 5% CO_2_. Infectious HCV JFH-1 was generated as previously described (Wakita *et al*., 2005). HCV JFH-1 infection of Huh7 cells was performed at a multiplicity of infection (MOI) of 0.1 as previously described (Wakita *et al*., 2005). Transfection of plasmids (pCMV-myc-Atg3, pCMV-myc-Atg5, pCMV-myc-Atg7) or small interfering RNAs (siRNAs) to Huh7 cells was carried out using Lipofectamine 3000 from Thermo Fisher Scientific China (Shanghai, China). The sequences of siRNAs in this study were as follows. Atg3-siRNA: TGTCATTCCAACAATAGAA; Atg5-siRNA: TTCATGGAATTGAGCCAAT; Atg7-siRNA: TTTGGGATTTGACACATTT; siRNAs and control siRNA (scrambled siRNA, the sequence is not available from the supplier) were synthesized from Qiagen China (Shanghai, China).

### MPA Treatment

Huh7 cells plated in 24-well plates were incubated in the presence of MPA (1 μg/mL-5 μg/mL) for 24 h–48 h. Tunicamycin-treated Huh7 cells were used as a positive control for inducing autophagy in Huh7 cells.

### Real-Time Reverse Transcriptase (RT) PCR

Total cellular RNA was extracted from Huh7 cells using Tri-Reagent and then subjected to reverse transcription using the reverse transcription kit from Takara company (Dalian, China). The real time RT PCR for the quantification of HCV RNA, autophagy-related genes, and glyceraldehyde-3-phosphate dehydrogenase (GAPDH) was performed with SYBR Green Master Mix (Takara, Dalian, China). The levels of GAPDH mRNA were used as an endogenous reference to normalize the quantities of target mRNA. To examine the levels of HCV RNA in culture supernatant, HCV RNA was extracted from 200 μL of supernatant by TRI-Reagent-BD Sigma-Aldrich China (Shanghai, China). Extracted RNA was then purified and digested with RNase-free DNase I (Qiagen, Shanghai, China). Copy numbers of HCV RNA were determined as previously described^49^. The primers were synthesized by Sangon Biotech Inc. (Shanghai, China) and the primer sequences are listed in Supplementary Table 1.

### PCR-Based Gene Expression Array

The autophagy -focused gene expression analysis was carried out according to the protocol provided by Qiagen (RT^2^ profiler PCR array kit, PAHS-084Z). Briefly, total cellular RNA was extracted from Huh7 cells treated with or without MPA for 24 h using SuperArray RT^2^ qPCR-Grade RNA Isolation Kit. The elimination of genomic DNA contamination and first strand cDNA synthesis were carried out using RT^2^ First Strand Kit. Autophagy-focused gene expression array was performed using RT^2^ Profiler TM PCR Array. Statistical analysis of data was performed with the Data-Analysis-Template provided by Qiagen.

### Western Blot

Total cell lysates from Huh7 cells were prepared using a radioimmune precipitation assay (RIPA) buffer (MultiSciences Biotech, Hangzhou, China) with 1% protease inhibitor cocktail. Protein concentrations were determined by DC protein assay kit. Western blot assay was carried out as previously described^49^,^50^. The primary antibodies used for western blot were as follows: rabbit-anti-β-actin (1:5000), mouse-anti-HCV core (1:1000), mosue-anti-LC3B (1:2000), rabbit anti-p62 (1:2000), mouse anti-Atg3 (1:1000), mouse anti-Atg5 (1:1000), mouse anti-Atg 7 (1:2500). The secondary antibodies were horseradish peroxidase-conjugated goat anti-rabbit IgG (1:10000) or goat anti-mouse IgG (1:5000), respectively. The immunoreactive bands were visualized by SuperSignal West Pico chemiluminescence substrate (Thermofisher, USA). The densitometric analysis of blots was performed by Image J software (National Institutes of Health, Bethesda, MD, USA). The values were normalized to those of control β-actin.

### Transmission electron microscopy

Transmission electron microscopy was utilized to detect the autophagy response in the HCV- infected or uninfected Huh7 cells treated with or without MPA. Tunicamycin treatment of the cells was used as positive controls. The cells were digested with trypsin and centrifugalized, and then immediately fixed with 2.5% glutaraldehyde/0.1 M sodium cacodylate. The cells were then postfixed with 1% osmium tetroxide, followed by dehydration with an increasing concentration gradient of ethanol and propylene oxide. The cells were then embedded with Epon618 and cut to ultrathin sections (50–60 nm) using an ultramicrotome (LKB-I). Images were examined utilizing a H7650 electron microscope at 80 kV after the samples were stained with 3% uranyl acetate and lead citrate.

### Statistical analysis

Where appropriate, data were expressed as mean ± standard deviation (SD) of at least triplicate experiments. Statistical significance was assessed by Student’s *t* -test. Statistical analyses were performed with Graphpad Instat Statistical Software. Statistical significance was defined as *p* < 0.05.

## Additional Information

**How to cite this article:** Fang, S. *et al*. Suppression of autophagy by mycophenolic acid contributes to inhibition of HCV replication in human hepatoma cells. *Sci. Rep.* **7**, 44039; doi: 10.1038/srep44039 (2017).

**Publisher's note:** Springer Nature remains neutral with regard to jurisdictional claims in published maps and institutional affiliations.

## Supplementary Material

Supplementary Information

## Figures and Tables

**Table 1:** Autophagy-related gene expression pattern modulated by MPA treatment in Huh7 cells.

Huh7 cells		HCV JFH1-infected Huh7 cells	
Gene	Fold Change	Gene	Fold Change
ATG3	−2.91	ATG3	−3.09
ATG5	−2.64	ATG5	−2.72
ATG7	−2.36	BECN1	−2.64
ATG16L2	−2.3	ATG7	−2.53
EIF4G1	−2.08	HSP90AA1	−2.2
GABARAP	−2.04	EIF4G1	−2.08
		HSPA8	−2.06
