# Supplementary material for: Suppression of autophagy by mycophenolic acid contributes to inhibition of HCV replication in human hepatoma cells
Source: Sci Rep. 2017 Mar 9;7:44039. doi: 10.1038/srep44039 (PMC5343675; doi:10.1038/srep44039)
Supplement: Supplementary Information [file srep44039-s1.pdf]

Suppression of autophagy by mycophenolic acid contributes to inhibition of HCV replication in human hepatoma cells

Shoucai Fang[1,2#], Jinming Su[1,3#], Bingyu Liang[1,2#], Xu Li[1,2], Yu Li[1], Junjun Jiang[1,2], Jiegang Huang[1,2], Bo Zhou[1,2], Chuanyi Ning[1,2], Jieliang Li[4], Wenzhe Ho[4], Yiping Li[5], Hui Chen[6], Hao Liang[1,2]* , Li Ye[1,2]*

Supplementary Figure 1

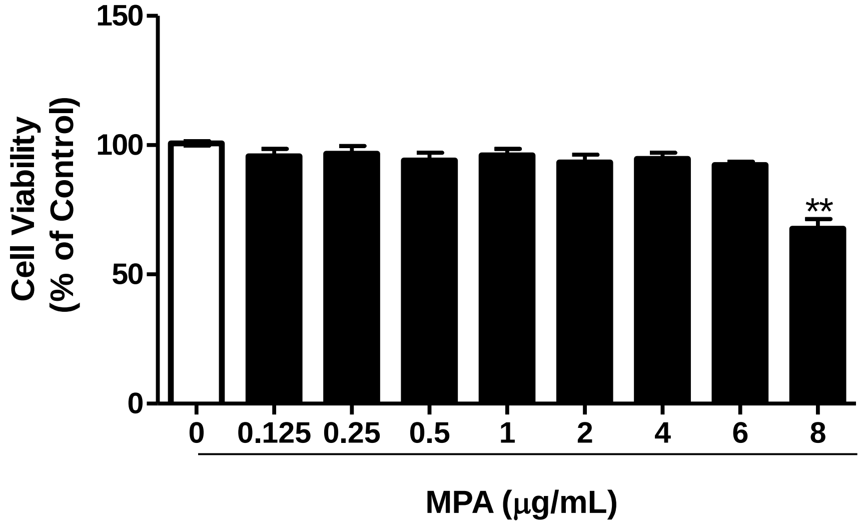

Supplementary Fig. 1. The cytotoxic effect of MPA on Huh7 cells.

Huh7 cells were treated with MPA at indicated concentrations for 72 h. The cell viability was assessed by MTS assay. The shown data are the mean ± SD of three independent experiments. The *p* value was calculated by Student's *t*-test (**, $p<0.01$).

Supplementary Figure 2

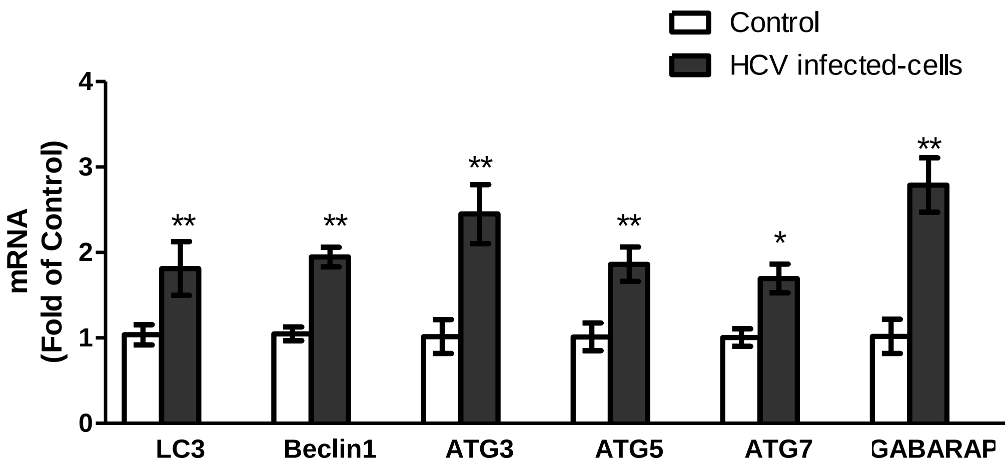

Supplementary Fig. 2. The effects of HCV infection on the expression of autophagy-related genes (ATGs) in Huh7 cells.

Huh 7 cells were infected with HCV JFH-1 at an MOI of 0.1. At 48 h postinfection. The cellular RNA was extracted and mRNA level of LC3, Beclin1, ATG3, ATG5, ATG7 and GABARAP was determined by real-time RT-PCR. With normalization to corresponding GAPDH mRNA level, the levels of ATGs are expressed as the fold of control (without HCV infectioin, which was defined as 1).The data shown are the mean ± SD of the results of three independent experiments. The *p* value was calculated by Student’s *t*-test (*, $p<0.05$, **, $p<0.01$).

Supplemental Table 1 The Primer sequences

Primer	Orientation	Sequence
HCV	Sense:	5’-RAYCACTCCCCTGTGAGGACC-3’
	Antisense:	5’-TGRTGCACGGTCTACGAGACCTC-3’
GAPDH	Sense:	5’-GGTGGTCTCCTCTGACTTCAACA-3’
	Antisense:	5’-GTTGCTGTAGCCAAATTCGTTGT-3’
LC3	Sense:	5’- AGCAGCATCCAACCAAAATC-3’
	Antisense:	5’-CTGTGTCCGTTCACCAACAG-3’
Beclin1	Sense:	5’-CCAGGATGGTGTCTCTCGCA-3’
	Antisense:	5’-CTGCGTCTGGGCATAACGCA-3’
ATG3	Sense:	5’- CCAACATGGCAATGGGCTAC-3’
	Antisense:	5’- ACCGCCAGCATCAGTTTTGG-3’
ATG5	Sense:	5’-TGGGATTGCAAAATGACAGA-3’
	Antisense:	5’-TTTCCCCATCTTCAGGATCA-3’
ATG7	Sense:	5’-CACTGTGAGTCGTCCAGGAC-3’
	Antisense:	5’-CGCTCATGTCCCAGATCTCA-3’
GABARAP	Sense:	5’-ACATTGCCTACAGTGACGAA-3’
	Antisense:	5’-TTTCAGTCCCTTCCAACTAC-3’
ATG16L2	Sense:	5’-TGGACAAGTTCTCAAAGAAGCTG-3’
	Antisense:	5’-CCTCAGTGCGACCAGTGAT-3’
EIF4G1	Sense:	5’-CCCGAAAAGAACCACGCAAG-3’

	Antisense:	5’-TTCCCCTCGATCCTTATCAGC-3’
HSP90AA1	Sense:	5’-AGGAGGTTGAGACGTTCGC-3’
	Antisense:	5’-AGAGTTCGATCTTGTTTGTTCGG-3’
HSPA8	Sense:	5’-ACTCCAAGCTATGTCGCCTTT-3’
	Antisense:	5’-TGGCATCAAAAACTGTGTTGGT-3’
